# “Occlusion” and “Articulation”

**Published:** 1904-11

**Authors:** Julio Endelman


### "OCCLUSION" AND "ARTICULATION."

The words "occlusion" and "articulation," so frequently quoted in dental literature, are often, we believe, erroneously referred to. The term occlusion, for instance, is not uncommonly made to imply the several and distinct relations of the teeth as witnessed in the act of mastication; whereas, in my conception, this term can refer to only one movement,—viz., to the closing of the jaws. Occlusion, therefore, refers to the normal relations of the inclined occlusal planes of the teeth when the jaws are closed (Angle). Articulation, on the other hand, is a name applied collectively to the relations of the lower teeth to the upper as exhibited in the several movements performed by the mandible during mastication.

Julio Endelman.

